# Climate for Consumptives

**Published:** 1882-06

**Authors:** 


### CLIMATE FOR CONSUMPTIVES.

SOME fifteen years ago, we published an article on the subject of localities of consumption. The general idea for which we contended was this, that warm climates hastened consumption; that an inseparable attendant of consumption, under all circumstances, was debility. The healthiest of us feel the debilitating effects of summer heats. And how an invalid is to be strengthened, by what debilitates a healthy man, we cannot understand. Consumptive people do not need the warm, damp, vapor-laden atmosphere of Cuba and Florida, but the cool, dry, still air of high latitudes. A man in consumption will more certainly get well in Greenland than in the West Indies.

From the details furnished from many sources, a member of the Massachusetts Medical Society has prepared a paper, conclusive of the fact, that all low and damp places originate and aggravate consumptive diseases, and that restoration and exemption must be found in cool and dry latitudes. And for similar reasons, sea voyages, and sea coast and lake shore and prairie localities have a pernicious effect upon all persons whose lungs are diseased. This subject is fully discussed under the head of Climate and Sea Voyages, in our book on "Bronchitis and Kindred Diseases."

